# Multidisciplinary care of pediatric obesity and its impact on sleep: a review

**DOI:** 10.3389/frsle.2025.1634185

**Published:** 2026-01-02

**Authors:** Ravali Inja, Christopher Cielo

**Affiliations:** Division of Pulmonary and Sleep Medicine, Children's Hospital of Philadelphia, Philadelphia, PA, United States

**Keywords:** sleep apnea, pediatrics, obesity, weight loss, obesity hypoventilation, multidisciplinary approach

## Abstract

Pediatric obesity has emerged as a significant global health issue with multifaceted consequences, including its impact on sleep health. Obstructive sleep apnea (OSA) and obesity hypoventilation syndrome (OHS) are among the serious sleep-related comorbidities in obese children, contributing to impaired quality of life, cognitive deficits, and cardiovascular risks. These conditions frequently coexist with other obesity-related complications such as insulin resistance, type 2 diabetes, hypertension, and non-alcoholic fatty liver disease (NAFLD). This review explores the importance of multidisciplinary care in addressing pediatric obesity, emphasizing early diagnosis, nutritional counseling, physical activity interventions, psychological support, and pharmacologic therapies such as glucagon-like peptide-1 (GLP-1) receptor agonists. The role of global trends, academic performance, and wellbeing clinics are also discussed. Although promising, the use of GLP-1s and surgical interventions in pediatrics remains constrained by limited data, particularly concerning their impact on sleep disorders. Further research is essential to clarify the long-term effects of GLP-1 receptor agonists and bariatric surgery not only on obesity and sleep-related comorbidities such as OSA and OHS, but also on cognitive function, psychosocial wellbeing, and overall health outcomes—thereby informing evidence-based, multidisciplinary approaches to pediatric obesity management.

## 1 Sleep disorders and associated comorbidities in pediatric obesity

The global prevalence of pediatric obesity has surged over recent decades due to a complex interplay of genetic predispositions, environmental influences, sedentary lifestyles, and socioeconomic factors. According to the Centers for Disease Control and Prevention (CDC), approximately 20% of children and adolescents in the United States are obese (CDC, 2022). Worldwide, the World Health Organization (WHO) estimates that over 340 million children and adolescents aged 5–19 were overweight or obese in 2016, with the numbers continuing to rise, especially in low- and middle-income countries undergoing rapid urbanization and dietary shifts (WHO, 2021).

Beyond well-documented risks like diabetes and cardiovascular disease, obesity in children significantly disrupts sleep health, notably through the development of sleep-disordered breathing conditions such as OSA and OHS. These disorders further exacerbate metabolic dysfunction and impair neurocognitive development, reinforcing a vicious cycle. In countries such as China, Brazil, and the United Kingdom, studies confirm similar associations between childhood obesity and poor sleep, emphasizing childhood obesity is a truly global crisis (Guo et al., 2020; de Carvalho et al., 2013).

### 1.1 OSA and OHS

Characterized by repeated episodes of upper airway obstruction during sleep, obstructive sleep apnea (OSA) leads to fragmented sleep and intermittent hypoxemia. OSA is particularly prevalent in obese pediatric populations. Verhulst et al. (2007) reported that up to 60% of obese children may present with clinical signs of sleep-disordered breathing, including habitual snoring, observed apneas, daytime sleepiness, and with 19% diagnosed with OSA. Structural airway abnormalities also contribute to OSA risk in this population. In their study, Arens et al. (2011) found that obese adolescents with OSA had significantly larger adenoids and tonsils compared to obese peers without OSA, suggesting that hypertrophic lymphoid tissue plays a critical role in airway obstruction among this group. The resulting nocturnal hypoxemia and sleep fragmentation contribute to insulin resistance, systemic inflammation, and elevated blood pressure. OSA has been found to be associated with reduced attention, executive function, and academic performance (O'Brien et al., 2004).

Recent advances in imaging and physiologic studies have provided deeper insight into the anatomical and neuromuscular mechanisms of OSA in adolescents. Schwab et al. (2015) demonstrated that obese adolescents with OSA have smaller upper airway caliber, greater lateral pharyngeal wall thickness, and increase tongue and soft palate volume compared to obese controls without OSA. These features increase airway collapsibility and resistance, even in the absence of craniofacial abnormalities (Bitners et al., 2020). Arens et al. (2011) further showed that during REM sleep, airway dynamics in obese adolescents with OSA are more unstable and susceptible to collapse due to reduced neuromuscular tone, particularly in the retroglossal and retropalatal regions. Marcus et al. (2017) emphasized that in adolescents, soft tissue enlargement and excess central fat contribute more significantly to OSA pathophysiology than lymphoid hypertrophy, which is more prominent in younger children.

Importantly, this anatomical vulnerability and sleep disruption contributes to a vicious cycle: disrupted sleep and recurrent hypoxia impair glucose metabolism, elevate cortisol levels, and promote leptin resistance, all of which increase appetite and visceral fat accumulation (Taheri et al., 2004). Sleep fragmentation also reduces daytime activity, impairs impulse control, and leads to fatigue-related overeating (Trosman and Trosman, 2017). Over time, this creates a self-reinforcing loop where obesity worsens sleep-disordered breathing, and sleep disorders accelerate weight gain and metabolic dysfunction. Marcus et al. (2017) highlight that adolescence is a particularly critical window when these physiologic changes are magnified by behavioral shifts—such as delayed bedtimes, increased independence in food choices, and screen exposure—further worsening both obesity and sleep health.

This bidirectional cycle not only affects energy balance but also impairs cognitive function, emotional regulation and school performance, compounding health and developmental risks. As such, disrupting this cycle through early identification and targeted intervention—whether through weight reduction, airway stabilization, improved sleep hygiene, or metabolic modulation—represents a key goal for future multidisciplinary strategies.

Defined by the triad of obesity, daytime hypoventilation (hypercapnia), and sleep-disordered breathing, obesity hypoventilation syndrome (OHS) presents with excessive daytime sleepiness, headaches, and fatigue. OHS is increasingly recognized in pediatric populations and may affect approximately 10–20% of obese children with sleep-disordered breathing (Lang and Bhammar, 2024). Most children with OHS also have coexisting OSA, and sleep-related hypoventilation often worsens during rapid eye movement (REM) sleep due to reduced ventilatory drive and upper airway muscle tone. Left untreated, OHS can lead to more severe complications than OSA alone, including pulmonary hypertension, right heart failure, and impaired quality of life. Marcus et al. (2012) emphasized that pediatric-specific clinical criteria for diagnosing OHS remain underdeveloped, complicating timely recognition and management. Globally, studies from the UK (Bonuck et al., 2015) and Southeast Asia (Ng et al., 2004) show similar patterns of OSA and OHS in obese youth, though access to diagnostic tools like polysomnography is often limited outside urban centers.

### 1.2 Insufficient sleep and overeating

Insufficient sleep in children—whether due to insomnia, poor sleep hygiene, and/or sleep disruption from OSA—is associated with increased appetite, reduced leptin and elevated ghrelin secretion, and a preference for calorie-dense foods (Taheri et al., 2004). This bidirectional relationship promotes weight gain and worsens metabolic outcomes such as insulin resistance, impaired glucose tolerance, elevated fasting insulin levels, dyslipidemia, and increased risk for type 2 diabetes and non-alcoholic fatty liver disease (NAFLD). Additionally, chronic sleep deprivation is linked to reduced impulse control, increased screen time, and emotional eating—factors that are exacerbated in high-stress or low-resource environments.

Insufficient sleep also amplifies the risk of overeating during evening hours, a pattern observed in both developed and developing countries (Weiss et al., 2010). Night eating syndrome (NES)—a disorder characterized by evening hyperphagia (consuming at least 25% of daily calories after dinner), frequent nocturnal awakenings with food intake, and morning anorexia—is more commonly studied in adults but has also been reported in adolescents. While less frequently diagnosed in younger children, NES-like behaviors such as night snacking and disrupted eating schedules are increasingly observed, especially in the context of obesity, mood disorders, and irregular sleep patterns. Irregular meal timing further contributes to circadian misalignment and weight gain, highlighting the importance of assessing sleep-wake and eating rhythms during pediatric obesity evaluation.

### 1.3 Psychological implications

Children with obesity often experience stigma, bullying, and reduced self-esteem, contributing to depression and anxiety. These psychological issues are frequently magnified when sleep disorders such as OSA or insomnia are present. Fatigue from poor or disrupted sleep can mimic or exacerbate symptoms of mood disorders, including irritability, low motivation, and cognitive sluggishness, complicating accurate diagnosis and treatment planning. Adolescents, in particular, may be less adherent to treatment recommendations for both sleep disorders and obesity—such as using CPAP devices, following dietary plans, or engaging in physical activity—due to embarrassment, low self-efficacy, or social withdrawal.

Studies in Europe and Australia show that children with obesity and sleep disorders report significantly lower health-related quality of life scores and higher rates of psychological distress than their normal-weight peers (Chaput et al., 2006).

### 1.4 Impact on school performance

Poor sleep quality due to OSA or insufficient sleep is associated with difficulties in concentration, memory, and academic performance. Trosman and Trosman (2017) demonstrated that children with OSA scored lower on standardized tests and exhibited more behavioral problems at school compared to their peers. These cognitive and behavioral challenges are often more pronounced in children with obesity, particularly those with coexisting OSA or obesity hypoventilation syndrome (OHS), due to the combined impact of sleep disruption, systemic inflammation, and metabolic dysregulation on brain function. Emerging evidence suggests that obese children with sleep-disordered breathing experience greater impairments in executive function and school performance than their normal-weight counterparts with similar sleep issues.

These findings are echoed globally, with research in Brazil and China indicating that sleep-disordered breathing significantly affects school engagement and academic achievement (de Carvalho et al., 2013; Guo et al., 2020). Given these associations, the presence of poor academic performance, daytime fatigue, or attention difficulties in children with obesity should prompt screening for underlying sleep disorders. Educational interventions and sleep health education in schools could serve as preventive measures. Additionally, teachers and school nurses may play a vital role in identifying children who appear chronically fatigued or are underperforming academically, facilitating earlier referral and diagnosis.

## 2 Interdisciplinary care for obesity and impact on sleep health

The management of obesity-related comorbidities including sleep disorders in children requires an interdisciplinary approach that includes physician specialists as well as professionals who can help ensure a healthy lifestyle, including eating and physical activity (Table 1). Global programs like the WHO's ECHO (Ending Childhood Obesity) framework support interventions across different cultural contexts, recommending national policies that promote healthy diets and physical activity from early childhood (WHO, 2016). Although ECHO identifies inadequate sleep as a key behavioral risk factor for obesity, it does not yet provide detailed strategies linking nutritional counseling to sleep improvement. This represents an important gap in pediatric obesity management and highlights the need for integrated approaches that consider sleep as both a consequence and potential target of lifestyle interventions. The integration of pediatric psychologists into multidisciplinary care teams is essential to screen for anxiety, depression, and disordered eating behaviors, and to provide tailored interventions that support adherence and emotional wellbeing early in the course of treatment.

**Table 1:** Role of interdisciplinary care team members and impact on obesity and sleep.

**Care component**	**Key roles and interventions**	**Impact on pediatric obesity**	**Impact on sleep health**
Nutritional counseling	Registered dietitians provide family-based, culturally sensitive dietary plans emphasizing portion control and nutrient-dense foods	Promotes weight reduction and improves metabolic health	Potential to improve sleep quality and reduce OSA severity
Physical activity programs	Exercise physiologists and physical therapists design age-appropriate activity plans; encourage ≥60 min/day moderate to vigorous activity	Reduces adiposity, improves cardio metabolic parameters	Enhances sleep duration and quality by regulating circadian rhythms
Sleep medicine specialists	Diagnose sleep disorders via polysomnography; recommend treatments like adenotonsillectomy or CPAP	Supports identification and management of OSA/OHS, often coexisting with obesity	Directly improves sleep-disordered breathing and sleep quality
Obesity medicine specialists	Oversee pharmacologic and lifestyle interventions including GLP-1 receptor agonists; monitor metabolic and psychological outcomes	Facilitates medically supervised weight management	Potential indirect benefits on sleep via weight reduction; emerging evidence on GLP-1 impact on sleep disorders
Psychological support	Pediatric psychologists screen for depression, anxiety, disordered eating; provide behavioral therapy to enhance adherence	Improves emotional wellbeing and supports sustainable lifestyle changes	Addresses sleep disruption related to mood and anxiety disorders
Health and Wellbeing Clinics	Provide integrated services: nutrition, psychology, physical therapy, and sleep evaluation under one roof	Enhance care coordinate and access, improve BMI, and psychosocial outcomes	Promote holistic improvement in sleep hygiene and management of sleep disorders
Surgical interventions	Bariatric surgery in select adolescents after thorough evaluation	Significant and sustained weight loss in severe obesity	Marked improved/resolution of OSA in many cases post surgery

### 2.1 Nutritional counseling

Nutritional counseling remains a cornerstone in the management of pediatric obesity. Registered dietitians provide structured, individualized plans that accommodate cultural preferences and family dynamics. Evidence supports family-based interventions as being more effective than those targeting the child alone (Epstein et al., 2007). Effective nutritional programs emphasize portion control, nutrient-dense food choices, and reduction of sugar-sweetened beverages. However, despite the well-documented benefits of dietary counseling on weight management and cardiometabolic health, its direct impact on sleep outcomes—such as sleep duration, quality, or the severity of sleep-disordered breathing—remains largely unstudied in children. While poor nutrition is known to affect sleep in adults, similar research in pediatric populations is sparse. Further investigation is needed to determine whether specific dietary interventions can improve sleep quality or reduce the burden of conditions like obstructive sleep apnea (OSA) or insomnia in obese youth.

### 2.2 Physical activity and exercise programs

Physical inactivity contributes significantly to pediatric obesity and sleep-disordered breathing. Regular physical activity improves body composition and reduces OSA severity by enhancing upper airway muscle tone and decreasing peripharyngeal fat (Dolezal et al., 2017). Children should engage in at least 60 min of moderate to vigorous activity daily (CDC, 2022). Programs that integrate activity into school curricula or community-based settings have shown success in diverse contexts, including the UK's “Daily Mile” initiative and Brazil's “Agita São Paulo” (Chillón et al., 2010). Tailoring interventions to individual needs—especially in children with limited mobility or comorbidities—requires collaboration with physical therapists and exercise physiologists.

Beyond these direct effects, there is a bidirectional relationship between physical activity and sleep quality. According to a study by Sampasa-Kanyinga et al. (2020), adolescents with shorter sleep duration engage in fewer daytime outdoor activities, which negatively impacts mental health through a stress susceptibility-recovery model. Poor sleep reduces motivation and energy levels, leading to decreased physical activity, while regular daytime activity, particularly outdoors, promotes better sleep by regulating circadian rhythms and reducing stress. This reciprocal interaction suggests that promoting physical activity may not only improve obesity and sleep-disordered breathing but also enhance sleep quality itself, creating a positive feedback loop that benefits overall physical and mental health in pediatric populations.

### 2.3 The role of sleep medicine specialists

Polysomnography remains the gold standard for diagnosing OSA and OHS in children. Pediatric sleep specialists play a key role in interpreting sleep studies, recommending treatment such as adenotonsillectomy or positive airway pressure, and evaluating efficacy. Adenotonsillectomy is first-line therapy for OSA in children, but is less effective in those with obesity, highlighting the need for additional OSA therapies and weight reduction strategies in tandem.

Multidisciplinary sleep clinics involving pediatric sleep medicine physicians, otolaryngologists, and behavioral sleep medicine specialists, when available, can provide comprehensive care to children and adolescents with obesity and a variety of sleep problems. Follow-up is crucial, as both weight status and sleep evolve with growth and development (Marcus et al., 2012).

### 2.4 Obesity medicine and health and wellbeing clinics

Pediatric obesity medicine specialists coordinate pharmacologic and lifestyle interventions while monitoring metabolic and psychological outcomes. With the recent approval of medications like liraglutide and semaglutide in pediatric populations, these specialists are increasingly central to care planning. In pediatrics, obesity medicine specialists are most commonly trained in pediatric endocrinology or general pediatrics, often with additional fellowship training or certification in obesity medicine. Some obesity specialists also have training in pediatric gastroenterology, and this background can be particularly helpful when nutritional and metabolic complexities are present. This multidisciplinary expertise allows them to tailor treatment plans that address the hormonal, metabolic, and behavioral aspects of pediatric obesity.

Health and wellbeing clinics—often based in hospitals or community health centers—offer interdisciplinary services including nutrition, psychology, physical therapy, and sleep evaluation. Children with obesity and sleep disorders frequently contend with depression, anxiety, poor self-esteem, and stigma. These psychological burdens not only diminish quality of life but also undermine adherence to treatment plans. Cognitive-behavioral therapy and other behavioral interventions have been effective in supporting sustainable lifestyle changes and emotional regulation, although research into their efficacy in children is more limited. Clinics modeled after the Yale Bright Bodies or Australian HIKCUPS (Hunter Illawarra Kids Challenge Using Parent Support) have demonstrated success in BMI reduction and psychosocial outcomes (Savoye et al., 2007; Collins, 2011).

GLP-1 receptor agonists have emerged as effective adjuncts for adolescents with severe obesity. The SCALE-TEEN trial demonstrated that liraglutide significantly reduced BMI when combined with lifestyle interventions (Kelly et al., 2020). Similarly, the STEP TEENS trial found that semaglutide achieved a 16.1% BMI reduction over 68 weeks (Weghuber et al., 2022).

Though GLP-1s have been shown to improve OSA severity in adults via weight loss, pediatric data are lacking. Future studies should explore their impact on sleep-disordered breathing in adolescents and long-term safety profiles.

### 2.5 Surgical interventions

Bariatric surgery may be appropriate for adolescents with severe obesity and comorbidities unresponsive to medical therapy. The Teen-LABS study reported significant weight loss and improvements in diabetes, hypertension, and OSA post-surgery (Inge et al., 2016). In adults, bariatric surgery has demonstrated sustained weight reduction and marked improvement or resolution of obstructive sleep apnea and other obesity-related sleep disorders, highlighting its potential benefits across age groups (Greenburg et al., 2009). However, surgery in pediatric patients requires rigorous psychological and nutritional screening, as well as long-term follow-up to monitor for potential nutrient deficiencies and mental health risks as well as the impact on sleep problems.

## 3 Discussion and conclusion

The intersection of pediatric obesity and sleep disorders constitutes a complex, multifactorial public health challenge with growing global significance. As this review highlights, conditions such as obstructive sleep apnea (OSA) and obesity hypoventilation syndrome (OHS) are prevalent among children with obesity, contributing to a cascade of physical, cognitive, and emotional impairments. In addition, poor sleep quality and insufficient sleep further aggravate weight gain by promoting hormonal imbalances, stimulating appetite and impairing glucose metabolism. This bidirectional relationship necessitates an integrated model of care that considers the interconnected roles of physical activity and sleep.

Physical activity enhances sleep quality and duration, which in turn supports weight regulation. Conversely, poor sleep leads to decreased energy and motivation for physical activity, creating a cycle that favors weight gain. Furthermore, obesity itself can impair sleep through increased risk of OSA and reduce physical activity levels because of fatigue, stigma, or biomechanical limitations. This cyclical relationship highlights the need for multidisciplinary interventions that concurrently address physical activity, sleep health, and obesity to disrupt these reinforcing pathways.

Globally, comorbid pediatric obesity and sleep disorders are not confined to the United States or high-income nations. Countries experiencing rapid urbanization and nutritional transitions report rising childhood obesity rates and associated comorbidities, but often lack the infrastructure to identify and manage conditions like OSA and insomnia. This global trend highlights the urgency of implementing accessible, culturally responsive interventions that address both metabolic health and sleep hygiene in diverse populations.

Multidisciplinary care is paramount to achieve optimal sleep health and manage obesity-related complications. Sleep medicine specialists are crucial for early identification of sleep-disordered breathing through tools like polysomnography and for initiating evidence-based treatments. Meanwhile, obesity medicine specialists guide comprehensive weight management strategies that include nutritional counseling, structured physical activity, and, when appropriate, pharmacological therapy with agents such as GLP-1 receptor agonists. Though data on GLP-1s in pediatric sleep disorders remain limited, early trials show promise in weight reduction—a critical step in mitigating OSA severity. Bariatric surgery, while effective in select cases, requires rigorous preoperative assessment and lifelong follow-up to manage its physiological and psychosocial implications.

Crucially, psychological health must be a core component of treatment. Academic underachievement is another downstream effect, driven by sleep fragmentation and associated neurocognitive deficits. Educators and pediatricians must collaborate to identify at-risk children and advocate for supportive school environments.

Health and wellbeing clinics offer a promising model by providing co-located, interdisciplinary services that promote holistic care. These clinics, especially when implemented in schools or community centers, improve care coordination and reduce barriers to access. Incorporating social determinants of health—such as food insecurity, neighborhood safety, and parental education—into assessment and intervention further ensures equitable outcomes.

In conclusion, pediatric obesity and sleep disorders are deeply intertwined conditions that demand early, comprehensive, and collaborative approaches. A framework integrating sleep medicine, obesity treatment, behavioral health, physical activity promotion, and family engagement provides the most effective pathway to long-term health. With the global rise in childhood obesity, there is a pressing need for scalable, evidence-based interventions and investment in pediatric sleep diagnostics, especially in low-resource settings. Future research should focus on the long-term efficacy and safety of pharmacologic treatments like GLP-1 receptor agonists in children, and on elucidating their impact on sleep architecture and neurodevelopmental outcomes. Addressing these challenges holistically will improve not only physical health but also the psychological resilience, academic success, and life trajectories of affected children worldwide.
